# Comparative genomics in acid mine drainage biofilm communities reveals metabolic and structural differentiation of co-occurring archaea

**DOI:** 10.1186/1471-2164-14-485

**Published:** 2013-07-17

**Authors:** Alexis P Yelton, Luis R Comolli, Nicholas B Justice, Cindy Castelle, Vincent J Denef, Brian C Thomas, Jillian F Banfield

**Affiliations:** 1 Department of Environmental Science, Policy, and Management, University of California, Berkeley, CA 94720, USA; 2 Earth Sciences Division, Lawrence Berkeley National Laboratory, Berkeley, CA, USA; 3 Department of Plant and Microbial Biology, University of California, Berkeley, CA 94720, USA; 4 Department of Earth and Planetary Sciences, University of California, Berkeley, CA 94720, USA; 5 Current address: Department of Civil and Environmental Engineering, Massachusetts Institute of Technology, Cambridge, MA 02139, USA; 6 Current address: Department of Ecology and Evolutionary Biology, University of Michigan, Ann Arbor, MI 48109, USA

**Keywords:** Metagenomics, Acid mine drainage, *Thermoplasmatales*, *Ferroplasma*, Iron oxidation, Comparative genomics

## Abstract

**Background:**

Metal sulfide mineral dissolution during bioleaching and acid mine drainage (AMD) formation creates an environment that is inhospitable to most life. Despite dominance by a small number of bacteria, AMD microbial biofilm communities contain a notable variety of coexisting and closely related *Euryarchaea*, most of which have defied cultivation efforts. For this reason, we used metagenomics to analyze variation in gene content that may contribute to niche differentiation among co-occurring AMD archaea. Our analyses targeted members of the *Thermoplasmatales* and related archaea. These results greatly expand genomic information available for this archaeal order.

**Results:**

We reconstructed near-complete genomes for uncultivated, relatively low abundance organisms A-, E-, and Gplasma, members of *Thermoplasmatales* order, and for a novel organism, Iplasma. Genomic analyses of these organisms, as well as *Ferroplasma* type I and II, reveal that all are facultative aerobic heterotrophs with the ability to use many of the same carbon substrates, including methanol. Most of the genomes share genes for toxic metal resistance and surface-layer production. Only Aplasma and Eplasma have a full suite of flagellar genes whereas all but the *Ferroplasma* spp. have genes for pili production. Cryogenic-electron microscopy (cryo-EM) and tomography (cryo-ET) strengthen these metagenomics-based ultrastructural predictions. Notably, only Aplasma, Gplasma and the *Ferroplasma* spp. have predicted iron oxidation genes and Eplasma and Iplasma lack most genes for cobalamin, valine, (iso)leucine and histidine synthesis.

**Conclusion:**

The *Thermoplasmatales* AMD archaea share a large number of metabolic capabilities. All of the uncultivated organisms studied here (A-, E-, G-, and Iplasma) are metabolically very similar to characterized *Ferroplasma* spp., differentiating themselves mainly in their genetic capabilities for biosynthesis, motility, and possibly iron oxidation. These results indicate that subtle, but important genomic differences, coupled with unknown differences in gene expression, distinguish these organisms enough to allow for co-existence. Overall this study reveals shared features of organisms from the *Thermoplasmatales* lineage and provides new insights into the functioning of AMD communities.

## Background

Until recently, very few genomes of archaea had been sequenced. As of 2012 there were only 233 archaeal genomes in the NCBI database compared to 3843 bacterial genomes. In part because of this bias, much less is known about archaeal evolution and physiology than that of bacteria. Of the sequenced archaeal genomes, most come from isolates from disparate environments and therefore tell us little about how archaeal populations co-exist within environments. Notable exceptions include isolates and draft genomes from metagenomic sequencing projects in hypersaline [1] and hot springs environments [2-5] and genomes of different strains of one gut methanogen [6]. Metagenomics allows us to examine the genomes of closely related archaea in the same community and make inferences about physiological differences that allow them to coexist. Spatial and temporal distributions of populations may be related to differences in geochemical conditions, in nutrients, or in other resources that different strains and species can utilize. Finally, if the intention is to isolate organisms with particular metabolic capacities, metagenomic insights can aid in the determination of the vitamins, nutrients, cofactors, and environmental conditions necessary for the growth of potential isolates.

A number of archaea of the *Euryarchaeal* order *Thermoplasmatales* have been described. This order currently comprises five genera: *Ferroplasma*, *Thermoplasma*, *Picrophilus*, *Thermogymnomonas*, and *Acidiplasma*. All of the isolates from this order are obligate or facultative aerobes and extreme acidophiles that were isolated from acidic, high sulfur environments. However, there is some phenotypic variation within this clade. The *Picrophilus* spp. are characterized by a single cell membrane surrounded by a surface layer, whereas the species in the other *Thermoplasmatales* genera have no cell walls. The *Thermoplasma* spp., *Picrophilus* spp., and *Thermogymnomonas acidicola* are moderate thermophiles with temperature optima around 60°C, whereas the *Ferroplasma* spp. and *Acidiplasma aeolicum* are mesophiles with temperature optima around 40° and 45°C respectively [7-15]. All of the isolates from the *Thermoplasmatales* order except for *Ferroplasma acidiphilum* are heterotrophs. All of the *Ferroplasma* spp. and *Acidiplasma* sp. are Fe-oxidizers and grow anaerobically via Fe respiration, whereas the *Thermoplasma* spp. are capable of S^0^ respiration.

In this study, we compare the near-complete genomes of the two *Ferroplasma acidarmanus* types, the isolate Fer1 sequence and the environmental Fer2 sequence, with newly annotated genomes of related organisms that we call A-, E-, G-, and Iplasma (APL, EPL,GPL, and IPL; NCBI accession numbers are reported in the Availability of supporting data section) [16,17]. These organisms coexist in biofilm communities sampled from within the Richmond Mine at Iron Mountain in Redding, California. Of these organisms, only Fer1 has been isolated [11]. Though some of the other genomes have been a part of previous metagenomic analyses [16-18], their gene content has not been fully examined. The gene annotations and microscopy reported here provide new insights into acid mine drainage (AMD) community function and genomic differentiation among these organisms that allows them to avoid competitive exclusion and thus co-occur.

## Results and discussion

### Phylogeny

**Figure 1:**
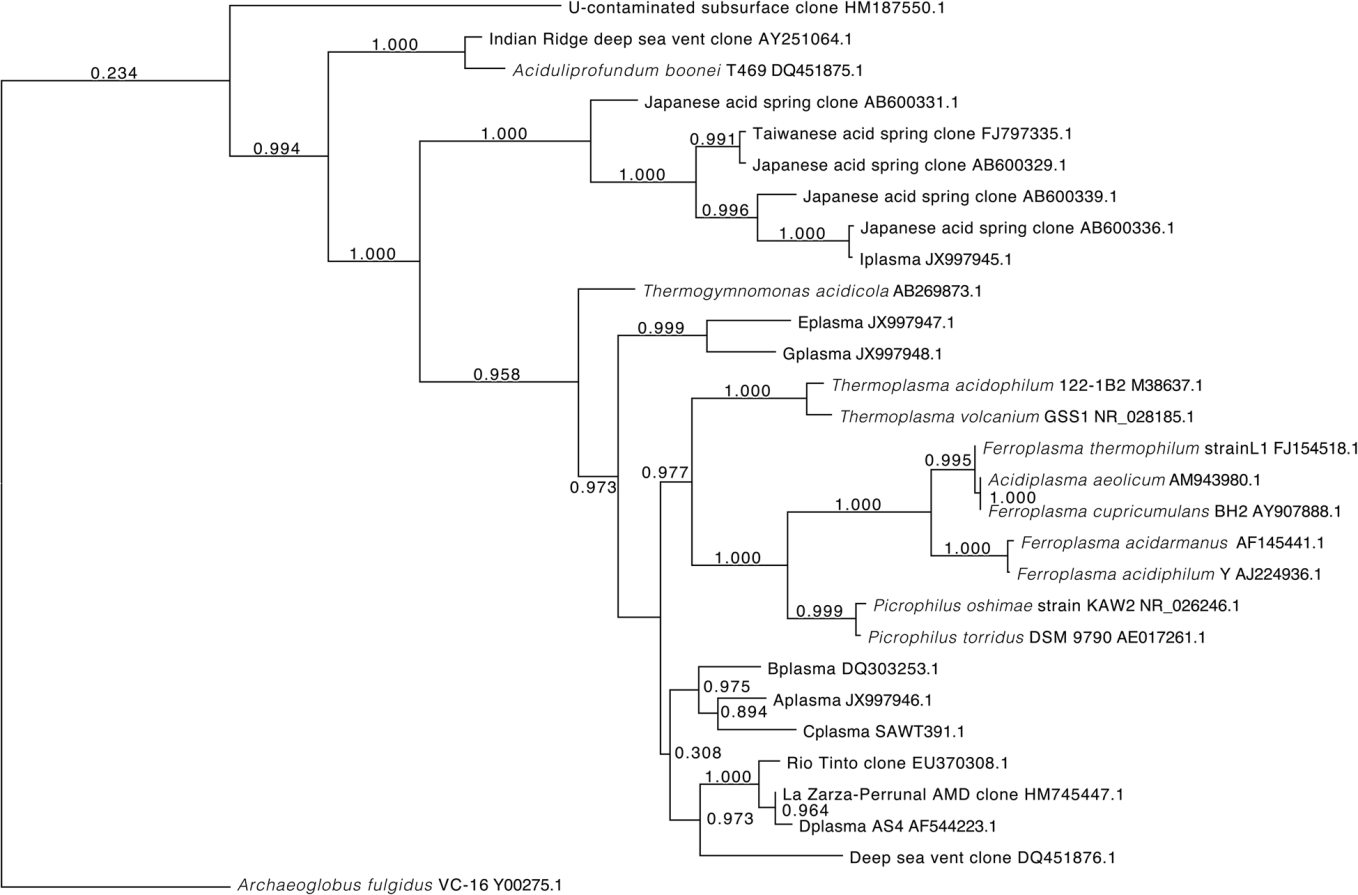
**16S rRNA tree indicating the possibility of a candidate class that includes Iplasma.***Ferroplasma acidarmanus* is Fer1 and Fer2. Bootstrap values are shown at branch splits. Gene start and stop positions and Genbank accession numbers are listed after organism names.

We previously published a phylogenetic tree of the 16S rRNA gene of the AMD plasmas [16,17]. Here we improve upon that tree with the addition of a number of new taxa. This tree illustrates that the Richmond Mine AMD plasmas form the following clades: A-, B-, and Cplasma, E- with G-plasma, Dplasma with a number of environmental clones, I-plasma with a number of environmental clones, and the *Ferroplasma* spp. with *Acidiplasma aeolicum*. All of the 16S rRNA gene sequences, other than those of Fer1 and Fer2 (which have identical sequences), share less than 97% nucleotide identity. The Iplasma gene is the most divergent, and it is almost certainly not a member of the order *Thermoplasmatales* or the class *Thermoplasmata* (Figure 1, Additional file 1, Additional file 2). We found evidence for this classification in the phylogenetic analysis for both 16S rRNA and ribosomal protein S15 genes, where Iplasma groups outside of the *Thermoplasmata* clade (Figure 1 and Additional file 3) as observed previously [16,17,19,20]. In the case of the 16S tree, Iplasma forms a monophyletic group with a number of environmental clones from acidic solfataric mud and acidic springs (Genbank) [21]. Because archaeal phylogeny is still unresolved, it is impossible to exactly determine the phylogeny of new taxa [22]. However, the branch length separating Iplasma and the *Thermoplasmata* organisms is greater than 0.25, supporting the separation of Iplasma into a new class of *Euryarchaea*. We previously suggested this in Justice *et al.*, 2012 [20], but the current study provides much more extensive evidence for this classification. The monophyletic clustering of Eplasma and Gplasma and that of A-, B-, and C-, and Dplasma on the 16S rRNA tree suggests that they belong to new genera of *Thermoplasmatales* (Figure 1, Additional files 1, 2). This finding is further supported by similar amino acid identities of shared orthologs from A-, E-, and Gplasma to the other *Thermoplasmatales* archaea (Additional file 4).

We examined a number of whole-genome measures of relatedness to further investigate evolutionary relationships. First, we identified the fraction of predicted orthologs in pairwise comparisons, and then determined their average amino acid identity. The normalization step involved dividing the number of orthologs by the average number of genes in the pair of genomes considered. Iplasma shares a lower percentage of orthologs, and a lower average amino acid identity with each of the other AMD plasma genomes than the other AMD plasma genomes share with each other (Additional files 4 and 5), consistent with a divergent phylogenetic placement. Fer1 vs. Fer2 has the highest amino acid identity (82%), as expected for closely related species. It was previously suggested that the genomes of Fer1 and Fer2 are different enough to merit classification as separate species based on analysis of recombination rates [23]. This result provides additional evidence supporting this claim, as Konstantinidis and Tiedje, 2005 found that approximately 95-96% amino acid identity corresponded to the 70% DNA-DNA hybridization species cut-off [24]. Eplasma and Gplasma are relatively closely related, as are Aplasma and Gplasma.

In addition to amino acid identity, we also looked at conserved gene order as a measure of evolutionary distance [16]. For each genome pair, we determined the number of syntenous orthologs and divided this by the number of shared orthologs. The Iplasma genome has the lowest synteny with the other AMD plasma genomes, Fer1 vs. Fer2 displays the highest synteny, followed by Eplasma vs. Gplasma (Additional file 6). The same trend holds true for another measure of synteny, the average length of syntenous blocks of genes in pairwise comparisons (Additional file 7). These whole-genome data support the tree topology and evolutionary distances assigned to the 16S rRNA genes in our phylogenetic analysis.

### General genome features

Genome features of the AMD plasma organisms, including the number of tRNA synthetases and ribosomal genes, are summarized in Yelton *et al.*, 2011 [16]. All of the genomes contain the full suite of tRNAs and most or all orthologous marker genes [16,25], consistent with a high degree of genome completeness (Additional file 8). Important metabolic and structural features of each genome are listed and illustrated in Table 1 and Additional file 9.

**Table 1:** General overview of metabolic differences within the AMD plasmas

**Function**	**APL**	**EPL**	**GPL**	**FER1**	**FER2**	**IPL**
**Aerobic metabolisms**						
Aerobic respiration	Y	Y	Y	Y	Y	Y
Fe oxidation (blue-copper protein)	Y	N	Y	Y	Y	N
Aerobic CODH	N	N	N	Y	Y	Y
Anaerobic CODH	N	N	N	N	Y	N
**Anaerobic metabolisms**						
Formate dehydrogenase	Y	Y	N	Y	Y	Y
Putative hydrogenase complex	Y	Y	Y	Y	Y	N
Fermentation to acetate	Y	Y	Y	Y	Y	Y
**Carbon catabolism**						
Glycolysis	Y	Y	Y	Y	Y	Y
Entner-Doudoroff pathway	Y	Y	Y	Y	Y	Y
Beta oxidation	Y	Y	Y	Y	Y	Y
Methylotrophy	Y	Y	Y	Y	Y	Y
**Biosynthesis**						
Cobalamin biosynthesis	N	N	N	Y	Y	N
Molybdopterin biosynthesis	Y	N	N	Y	Y	Y
Histidine synthesis	Y	N	Y	Y	Y	N
Leucine/Isoleucine synthesis	Y	N	Y	Y	Y	N
Glyoxylate shunt	N	Y	N	N	N	N
**Motility**						
Flagella	Y	Y	N	N	N	N
Chemotaxis	N	N	N	N	N	N
**Toxic metal resistance**						
Arsenic resistance	Y	Y	Y	Y	Y	Y
Copper resistance	Y	Y	Y	Y	Y	Y
Mercury resistance	Y	Y	Y	Y	N	Y
**Structure/Motility**						
S-layer	Y	Y	Y	N	Y	Y
Ether-linked lipids	Y	Y	Y	Y	Y	Y
Cellulose/cell wall polysaccharides	N	N	N	N	N	N
Pili	N	Y	Y	N	N	Y

**APL is Aplasma. EPL is Eplasma. GPL is Gplasma. FER1 and FER2 are *Ferroplasma acidarmanus *type I and type II. IPL is Iplasma.** Y indicates that the pathway is found in the genome, whereas N indicates that it is not.

### Unique genomic island in G-plasma

**Figure 2:**
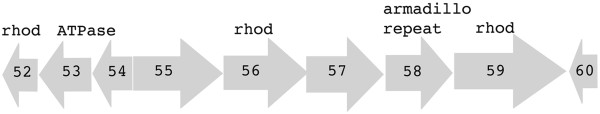
**Cluster of unique genes in Gplasma.** Arrows are proportional to the length of each gene and indicate its direction of transcription. The gene numbers are shown inside the arrows. All genes are from contig number 13327. Motif and domain-based annotations are shown above the arrows. Genes with no annotations are hypothetical proteins. Rhod indicates a rhodanese-like domain.

A genomic island of potential importance was identified in the Gplasma genome. It consists of a block of nine genes that have virtually no orthologs in any of the other *Thermoplasmatales* genomes and is made up primarily of proteins of unknown function (Figure 2, Additional file 10). All nine of the proteins are represented in a whole community proteomic dataset reported previously [26], and three are among the most highly detected proteins of this organism in that dataset. The motifs and domains identified suggest that a number of these proteins are membrane associated, including a protein containing an AAA + FtsH ATPase domain (gene number 13327_0053) (found in a membrane-integrated metalloprotease [27]), a protein containing six transmembrane motifs and a signal peptide (13327_0056), and another with fourteen transmembrane motifs and a signal peptide (13327_0059). Additionally, three of these proteins include a rhodanese-like domain possibly involved in phosphatase or sulfurtransferase activity and another contains an armadillo repeat region, often used to bind large substrates such as peptides or nucleic acids (13327_0058).

The absence of any orthologs to this block of hypothetical proteins in other *Thermoplasmatales* genomes is a strong indication that it may have been acquired by horizontal gene transfer. Many flanking genes have syntenous orthologs in other closely-related genomes. However, the lack of GC skew in the nucleotide signature of these genes suggests that the transfer event was not recent or that the donor had a similar GC content to Gplasma.

### Cell wall biosynthesis and imaging

**Figure 3:**
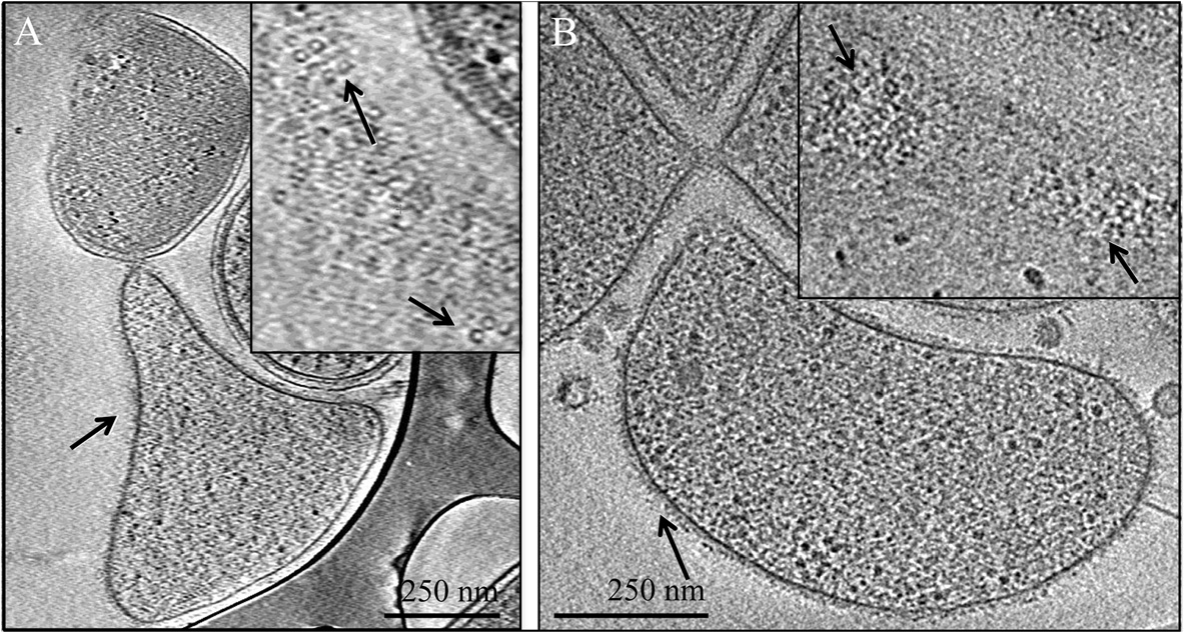
**Cryo-EM of surface-layer on an AMD plasma cell from the Richmond Mine.** Insets show a higher magnification. Arrows point to putative surface-layer proteins. Panel **A** and panel **B** show evidence of proteinaceous surface layers in two different cells collected from the Richmond Mine AMD.

*Thermoplasmatales* cells are generally bounded by a single membrane, except for two *Picrophilus* species that have a single membrane surrounded by a surface-layer (S-layer) [13]. We characterized archaeal-rich biofilm communities via cryo-electron microscopy and identified surface layers on many single membrane bound cells (Figure 3, Additional file 11). Thus, we looked for the genes needed for surface layer structural proteins and their post-translational modifications (i.e., N-glycosylation). We found putative S-layer genes in all of the AMD plasma genomes (except Fer1) that are homologous with the predicted *P. torridus* S-layer genes (Additional file 12) [28], but found no homology to the predicted S-layer genes in their next closest relative, *Acidiloprofundum boonei*[29]. We also found genes potentially involved in archaeal S-layer protein N-glycosylation. Of particular interest were homologs to the AglD and AglB genes of *Haloferax volcanii*, which have been shown to be essential to S-layer protein N-glycosylation in that organism [30]. Many of the Iplasma S-layer-related genes occur in a cluster, and several have conserved gene order in distant relatives, including several enzymes that attach sugars to a dolichol that might serve as a membrane anchor for the formation of an oligosaccharide during N-glycosylation. The Iplasma genome contains a gene cluster syntenous with distant relatives that encodes all of the proteins in the ADP-L-glycero-β-D-manno-heptose (AGMH) biosynthesis pathway (Additional file 12). AGMH is attached to S-layer proteins in gram-positive bacteria [31-33], suggesting that this may be involved in S-layer glycosylation in Iplasma as well. Finally, in the same genomic region genes are found for the biosynthesis of GDP-L-fucose, a glycoprotein component, and dTDP-L-rhamnose, a lipopolysaccharide component, indicating that these may make up part of the AMD plasma S-layer polysaccharides.

### Energy metabolism *(a) iron oxidation*

Ferric iron produced by biotic iron oxidation drives metal sulfide mineral dissolution, and thus iron oxidation is one of the most important biochemical processes that occurs in acid mine drainage systems [34-36]. In order to assess which of the AMD plasmas were involved in this process, we looked for potential iron oxidation genes via BLASTP. Based on this analysis, Aplasma and Gplasma contain homologs to rusticyanin, a blue-copper protein implicated in iron oxidation in *Acidithiobacillus ferrooxidans* (Additional file 12) [37]. The *Acidithiobacillus ferroxidans* rusticyanin can complex with and reduce cytochrome c in that organism [38-41], is upregulated during growth on ferrous iron [40-47], and is believed to be essential to iron oxidation [48]. Allen *et al.*[49] inferred that a related blue-copper protein, sulfocyanin, is involved in iron oxidation in *Ferroplasma* spp. (e.g. Fer1), and Dopson *et al.* provided proteomic and spectrophotometric evidence that support this inference [50]. The Fer2 genome contains a sulfocyanin homolog, whereas E- and Iplasma do not appear to have a rusticyanin or a sulfocyanin gene, suggesting that they are not iron oxidizers.

Additional evidence for the function of these genes was found in their inferred protein structure. All of the AMD plasma blue-copper proteins (BCPs) contain the characteristic type I copper-binding site, consisting of two histidines, one cysteine, one methionine and a cupredoxin fold, identified by a 7 or 8-stranded β-barrel fold [51-53] (Additional file 13). However, the AMD plasma BCPs differ in their conservation of motifs identified by Vivekanandan Giri *et al.* in sulfocyanin and rusticyanin [54]. The Fer1 and Fer2 BCPs include one recognized sulfocyanin motif, FNFNGTS, as well as imperfect conservation of the motifs identified in both sulfocyanin and rusticyanin (Additional file 14). Conversely, the Aplasma and Gplasma blue-copper proteins do not contain any of the conserved sulfocyanin-specific motifs. Instead, they contain imperfect matches to the rusticyanin-specific motif. These results are consistent with the inferences made based on homology alone in that they suggest that Fer1 and Fer2 BCPs are sulfocyanins and that A- and Gplasma BCPs are rusticyanins.

Phylogenetic analysis was carried to confirm the original homology-based annotations of the AMD plasma BCPs and to look for evidence of horizontal gene transfer. The phylogenetic tree groups the Aplasma BCP gene with the rusticyanins, whereas the Fer1 and Fer2 genes group with the sulfocyanins (Additional file 15). Interestingly, the Gplasma gene is so divergent that it does not consistently group with the other iron-oxidation blue-copper proteins. Its divergence seems to stem from two more β-strands than most of the other rusticyanin-like proteins (Additional file 13). The tree also provides evidence for the horizontal transfer of both sulfocyanin and rusticyanin genes. Related rusticyanin-like genes are found in the *Gammaproteobacteria* and in a variety of *Euryarchaea*. Similarly, closely related sulfocyanin-like genes are found in *Euryarchaea* and *Crenarchaea*.

Tyson *et al.* hypothesized that the sulfocyanin found in the Fer1 genome forms part of an iron-oxidizing SoxM-like supercomplex, similar to the one involved in sulfur oxidation in *Sulfolobus acidocaldarius*[55-57]. The *S. acidocaldarius* SoxM supercomplex contains a BCP, a cytochrome b and a Rieske iron sulfur protein. In *S. acidocaldarius* the sulfocyanin functions much like the cytochrome c in the complex III/cytochrome bc complex used during iron oxidation (and aerobic respiration) in *A. ferrooxidans*[58]. The results presented here further support Tyson’s hypothesis in that both the cytochrome b and rieske Fe-S protein subunits of the hypothetical SoxM-like complex were identified in all AMD plasma genomes. None of the genomes contain homologs to any of the other genes in the *A. ferrooxidans rus* operon [42,59,60].

In general, the absence of blue-copper proteins suggests that E- and Iplasma lack the Fe-oxidation capability entirely, whereas the other AMD plasmas utilize two different pathways to carry out this metabolism. It is possible that E- and Iplasma do have blue-copper proteins in their genomes because gaps remain in their assemblies, but we took steps to rule out this possibility (see Methods section). Because Fe(II) is an abundant electron donor in the AMD environment, this observed genetic variation in Fe oxidation potential may be important in niche differentiation.

### Energy metabolism *(b) carbon monoxide dehydrogenase*

The Iplasma, Fer1 and Fer2 genomes encode genes for a possible carbon monoxide dehydrogenase, (CODH) (Additional file 12), including genes for all three subunits of the CoxMLS complex. Recent research suggests that aerobic CO oxidation may be a widespread metabolism among bacteria [61]. Thus, it is a conceivable metabolism for organisms in AMD systems. In fact, it may be a good source of carbon or energy in the Richmond Mine, where up to 50 ppm of CO has been measured in the air (M. Jones, personal communication 2011).

A phylogenetic tree of the catalytic subunits of CODH indicates that all but one of the AMD plasma complexes is more closely related to the aerobic type than the anaerobic type (Additional file 16). The active site encoded by these genes also suggests that they are aerobic CODH proteins closely related to the form II CODH, which has the motif: AYRGAGR (Additional file 17) [61,62]. This enzyme can be used to make CO_2_ either for C fixation or to make reducing equivalents. The AMD plasma genomes do not contain any of the genes for the known archaeal C fixation pathways. Based on these observations, we hypothesize that these CODH proteins are used solely to make electrons available for aerobic respiration. However, it is possible that they use a novel C fixation pathway that incorporates this CODH [63].

Interestingly, our CODH phylogenetic tree suggests that there is another AMD plasma gene that encodes a Ni-CODH, Fer2 scaffold 31 gene 47. Ni-CODHs are anaerobic and reduce CO_2_ to CO. This enzyme is generally involved in C fixation via the Wood-Ljungdahl pathway, the genes for which are not found in the AMD plasma genomes. Thus, this gene may be involved in a novel carbon fixation pathway in Fer2. Additional evidence for the annotation of this gene as a Ni-CODH is provided in its structural alignment with known Ni-CODH proteins (Additional file 18), and by the annotation of a neighbor gene as a Ni-CODH maturation factor (Additional file 12). As a whole, the genomic evidence suggests CO oxidation capacity among Fer1, Fer2, and Iplasma and a potential for CO reduction in Fer2.

### Energy metabolism *(c) aerobic respiration*

Fer1 and *T. acidophilum* are known to be facultative anaerobes [11,64-66], whereas *T. volcanium* and *P. torridus* are aerobes. Therefore, it is not surprising that all of the Richmond Mine AMD plasmas have the capacity for aerobic respiration and catabolism of organic compounds via two glucose catabolism pathways, pyruvate dehydrogenase, the TCA cycle and an aerobic electron transport chain (Additional file 12). Some AMD plasma genes in the aerobic electron transport chain have been observed in proteomic analyses as previously reported by Justice *et al.*, 2012 [20].

The AMD plasmas’ electron transport chains are similar to that of other archaea in that they do not contain all of the subunits of the NADH ubiquinone-oxidoreductase complex [67]. All of the AMD plasmas except Aplasma are missing the NuoEFG subunits found in the bacterial type complex I and instead have the subunits found in the archaeal-type complex I, NuoABCDHIJKLMN. Fer2 is missing NuoIJKLM most likely because the genes for this complex are found at the end of an incomplete contig. Eplasma, Gplasma and Fer1 maintain the Nuo gene order found in a number of other archaea including, *Halobacterium sp.*, *Sulfolobus solfataricus*, and *T. acidophilum*[68]. All contain succinate dehydrogenase complex genes (Additional file 12). In the case of A-, E-, and Gplasma, the complex is missing SdhD, and many of the SdhC genes have annotations with low confidence. This finding is congruent with previous research that shows that the genes for the membrane anchor subunits of the complex are poorly conserved in both bacteria and archaea, possibly due to low selective pressure [69]. As mentioned previously in section (v)(a), the AMD plasmas have genes homologous to several predicted archaeal complex III/cytochrome bc complex genes (Additional file 12).

Archaeal-type aerobic terminal oxidases include cytochrome c oxidases (CCOs) and cytochrome bd oxidases. Genes for the cytochrome bd complex are found in *P. torridus*, *T. acidophilum* and *T. volcanium*[70]. All of the AMD plasma genomes contain the two genes for this complex. They also all contain the two essential genes for the archaeal heme-copper oxidase/CCO complex (subunit I and II) [70], and we confirm that subunit II contains the Cu-binding motif generally found in CCOs [71] (Additional file 19). Like the other CCO genes in *B. subtilis* and *E. coli,* the two cytochrome c genes in the AMD plasmas occur in a gene cluster with a protoheme IX farnesyltransferase, required for synthesis of the heme type used in aa(3) type CCOs [72]. The subunit II gene shares a high amino acid identity with several oxidases of this type, further indicating an aa(3) type CCO (Additional file 20).

Archaea use A-type ATP synthases to generate ATP from an electrochemical gradient. All of the AMD archaeal genomes contain the AhaABCDEFIK genes that comprise this complex in *Methanosarcina mazei*, although they are missing an ortholog to AhaG. All but Eplasma and Iplasma contain a putative AhaH gene. AhaG is also absent in *T. acidophilum*, indicating that it may not be necessary for ATP synthesis in these organisms.

### Energy metabolism *(d) alternative electron acceptors*

In addition to aerobic respiratory capabilities, some *Thermoplasmatales* organisms are able to respire anaerobically [66]. Anaerobic reduction of S^0^ or sulfur ions could allow archaea in AMD systems to survive under anoxic conditions deep inside floating biofilms or in sunken biofilms and sediment, where many sulfur compounds are present [73]. The Iplasma genome contains several genes that are homologous to *asrA* and *asrB*, known sulfite reduction protein genes (13606_0515 and 13606_0514). These proteins comprise two of the three subunits of the AsrABC dissimilatory sulfite reductase complex found in *Salmonella typhimurium*[74]. However, the Iplasma genome does not contain the AsrC subunit, which contains the siroheme-binding motif and thus is thought to contain the active site for sulfite reduction. As the Asr proteins are not well characterized in many organisms, it is possible that these genes are misannotated. Synteny-based annotation ties these two genes to an adjacent FdhF formate dehydrogenase alpha subunit gene, indicating a possible involvement of these genes in formate dehydrogenase activity. In fact, one of these genes is structurally related to the HycB hydrogenase 3 Fe-S protein formate dehydrogenase subunit based on CBLAST against the NCBI protein structure database. Additional protein modeling suggests that one of the proteins in Iplasma could be a subunit of the formate dehydrogenase complex (Yelton, Zemla, and Thelen; unpublished observation). Thus, we suggest that these two proteins are functionally related to formate dehydrogenase in Iplasma.

Interestingly, the Iplasma genome contains homologs to all of the genes overexpressed under anaerobic conditions for *T. volcanium* as well as all of the genes overexpressed or over-transcribed under anaerobic conditions for *T. acidophilum* (except for their predicted sulfur respiration gene Ta1129) in two previous studies [75,76] (Additional file 21). The other AMD archaea also share most, but not all, of these genes. Although there is no direct genomic evidence for anaerobic respiration, novel anaerobic respiratory pathways are possible. In fact, there is evidence that Fer1 can grow via anaerobic Fe(III) reduction [64], and enrichment cultures of Fer1 and Aplasma reduce iron [20].

### Energy metabolism *(e) heterotrophy*

Chemolithoautotrophy is a common lifestyle in AMD communities (e.g., of *Leptospirillum* spp.) [77]. However, the *Thermoplasmatales* archaea are mostly heterotrophs (only *F. acidiphilum* has been shown to have any autotrophic capability [10]). The AMD plasma genomes encode genes for a wide variety of heterotrophic metabolisms, both aerobic and anaerobic. The AMD plasmas have the genes necessary for energy generation via catabolism of organic compounds, including fatty acids, sugars, starch, and glycogen, but not refractory organic matter such as cellulose (Additional file 12).

All of the AMD plasmas have genes for sugar and polysaccharide catabolism, including glucoamylase genes required to break down starch and alpha-amylase genes for glycogen catabolism into glucose and dextrin. They have the conventional Embden-Meyerhoff (EM) glycolytic pathway (Additional file 12). Moreover, they also have the genes for the non-phosphorylative Entner-Doudoroff (NPED) pathway for glucose degradation also found in a number of (hyper)thermophilic archaea, including *T. acidophilum*, *P. torridus*, *S. solfataricus*, *Sulfolobus acidocaldarius*, *Sulfolobus tokodai* and *Thermoproteus tenax*[78-81]. The AMD plasma genomes contain homologs to all of the genes in this pathway, including a homolog to the proven *P. torridus* KDG aldolase [82]. Thus, the AMD plasmas are similar to their *Thermoplasmatales* relatives, all of which have genes homologous to those of both the EM and NPED pathways. Previously published proteomic data indicates that all of the AMD plasma organisms express some of the genes in these two pathways [20].

Another potential carbon source for the AMD plasmas is lipids from lysed cells. All of the AMD plasma genomes contain a full set of homologs to the genes for the aerobic fatty acid oxidation pathway from *E. coli* (Additional file 12). Because many of the proteins in this pathway are acyl-CoA dehydrogenases, which are known to have undergone frequent gene duplication and horizontal transfer events [83], it is difficult to discern which role each gene plays in fatty acid degradation. However the number of β-oxidation-related annotations suggests that the AMD plasmas are capable of fatty acid breakdown, and many of the proteins from this pathway have been identified by proteomics [20].

Interestingly, the AMD plasmas have the genetic capacity to catabolize one-carbon compounds such as methanol. All except for Gplasma have several genes for subunits of a formate dehydrogenase. These genes were previously discussed by Yelton et al. [16], and a number are found in gene clusters with biosynthesis genes for their specific molybdopterin cofactor. We find that a formate hydrogen lyase complex gene cluster is evident in the Fer1 genome, as previously noted by Cárdenas *et al.*[63], but we also find a cluster of orthologous genes in Eplasma and Gplasma. It is possible that Fer1 is capable of the chimeric pathway of carbon fixation involving the formate hydrogen lyase described by Cárdenas *et al.*[84] (See section (vi) for further discussion of the putative group 4 hydrogenase *hycE* gene in this cluster). Eplasma also has the genes necessary for this pathway, but all of the other AMD plasma genomes are missing either the formate hydrogen lyase genes or the formate dehydrogenase subunit genes. Thus, we surmise that the AMD plasma formate dehydrogenases are primarily involved in an oxidative pathway for methanol methylotrophy (i.e., methanol degradation to formaldehyde, formaldehyde to formate, and formate oxidation to CO_2_). The AMD plasmas have homologs to all of the enzymes in this pathway, including the enzyme used by all thermotolerant methanol-oxidizing bacteria, a NAD-linked methanol dehydrogenase [85] (Additional file 12). Among the AMD plasmas, only Iplasma appears to have the genes necessary for the ribulose monophosphate cycle, which is commonly used for carbon assimilation from formaldehyde [85]. None of the genomes contain the genes necessary for the other known formaldehyde assimilation pathway, the serine cycle. As Fer1 has been shown to produce methanethiol during cysteine degradation [86], any methanol in the AMD biofilm may be a product of methanethiol catabolism.

### Energy metabolism *(f) fermentation and the use of fermentation products*

AMD archaea are typically more abundant in thick, mature AMD biofilms [87] where they may encounter anoxic microenvironments [73]. Thus, we looked for potential fermentation genes in their genomes. They all have the genes for fermentation of pyruvate to acetate found in *Pyrococcus furiosus* and a number of other anaerobic fermentative and aerobic archaea [88-91] (Additional file 12). This pathway is unique in that it converts acetyl-CoA to acetate in only one step, with an ADP-forming acetyl-CoA synthetase. It is the only phosphorylating step of pyruvate fermentation via the NPED pathway. Previously this enzyme had been detected in hyperthermophilic and mesophilic archaea as well as some eukaryotes [91]. In anaerobic archaea this enzyme is involved in fermentation, whereas in aerobic archaea it makes acetate that is then catabolized via aerobic respiration [92]. The AMD plasmas have the genes necessary for fermentation to acetate under anaerobic conditions and for acetate respiration under aerobic conditions via an acetate-CoA ligase or the reversal of the direction of the acetate-CoA synthetase.

### Putative hydrogenase 4 genes

Several AMD plasma genomes contain a number of genes that group with the putative group 4 hydrogenases according to phylogenetic analysis (Additional file 22). A group 4 hydrogenase complex and formate dehydrogenase comprise the formate hydrogen lyase that catalyzes non-syntrophic growth on formate and production of H_2_ in hyperthermophilic archaea (*Thermococcus onnurineus*) [93,94]. The putative group 4 hydrogenases, though closely related to the group 4 hydrogenases, lack the two conserved hydrogen and Ni-binding motifs that are thought to be necessary for H_2_ formation [94,95], possibly indicating some other function.

### Toxic metal resistance

The Richmond Mine solutions contain extremely high (mM) concentrations of arsenic, cadmium, copper, and zinc [96]. Genomic evidence indicates that the AMD plasmas utilize multiple strategies to protect themselves from these elements, such as oxidation/reduction to less toxic forms and efflux (Additional file 12) [8,97]. All of the AMD plasmas have at least two genes from the arsenic resistance (*arsRABC*) operon. Only Gplasma has all of the genes in the operon, but Fer1 has previously been shown to have resistance to both arsenate and arsenite, despite lacking the arsenate reductase [97]. All of the AMD plasmas except for Fer2 have two of the genes in the mercury resistance operon (*merTPCAD*), *merA* and *merP* (mercuric reductase and the mercuric ion-binding protein, respectively). All of the genomes also contain some putative copper resistance genes in the *copABCD* operon or the *copYBZ* loci, identified previously in Fer1 [98]. Specifically they all have homologs to *copB*. This gene has been shown to be involved in copper sequestration as a copper resistance strategy in *Pseudomonas syringae*[99]. The heavy metal transporter genes found in the AMD plasma genomes group into two different clades in a phylogenetic tree of metal resistance P-type ATPases. All of the genomes except for that of Iplasma contain two types of metal resistance transporters according to this phylogenetic analysis, a Cu/Ag transporter related to *copA* or *copBZ* and a Zn/Cd transporter related to *cadA*.

### Biosynthesis

Because the AMD plasmas live in dense biofilms, they could potentially benefit from biomolecules (cofactors, amino acids, etc.) provided by other organisms .We previously demonstrated a lack of genes for *de novo* cobalamin biosynthesis in A-, E-, G-, and Iplasma [16]. Here we examined the AMD plasma genomes for other biosynthetic pathways.

### Biosynthesis *(a) glyoxylate shunt*

Only Eplasma has the genes for the glyoxylate shunt, a pathway closely related to the TCA cycle that allows the use of organic compounds that are degraded to acetyl-CoA (i.e. fatty acids) for biosynthesis (Additional file 12). One of the proteins encoded in this pathway, the malate synthase, has been detected in proteomic analyses [20].

### Biosynthesis *(b) amino acid synthesis*

The *Thermoplasmatales* archaea exhibit differential abilities to synthesize amino acids, suggesting that some of them rely more heavily on organic compound uptake than others. The genomes of E-, G- and Iplasma do not contain most of the histidine synthesis pathway genes. Eplasma and Iplasma also lack many of the genes necessary for the valine and (iso)leucine synthesis pathway (Additional file 12). They are also among the subset of organisms that do not make their own cobalamin [16]. This group of organisms may rely on amino acid and cobalamin scavenging to avoid the energetic costs of *de novo* synthesis.

### Biosynthesis *(c) trehalose biosynthesis*

Compatible solutes allow organisms to maintain osmotic balance under high salt conditions or to protect against heat shock and cold shock [100]. A number of archaea make organic solutes for this purpose. *T. acidophilum* and a number of *Sulfolobales* archaea have been shown to produce trehalose as a compatible solute. In these organisms it has also been suggested that it is used to thermostabilize macromolecules and as a carbon storage molecule [100]. All of the AMD plasmas except for Iplasma have the genes necessary for trehalose biosynthesis from maltose (Additional file 12). The monophyletic group of A-, E-, and Gplasma also has the genetic potential for trehalose synthesis from glycogen.

### Motility

Motility can provide a competitive advantage for archaea in aquatic environments by allowing them to colonize new sites and move across environmental gradients. To determine potential for motility, we looked for flagellar, chemotaxis and pili genes in the AMD plasma genomes.

Both the A- and Gplasma genomes contain the full flagella *flaBCDEFGHIJ* operon found in *Methanococcus voltae*[101-103] and *Halobacterium salinarum*[104] (Additional file 12). Thus, these organisms are predicted to be motile, yet they lack identifiable chemotaxis genes.

**Figure 4:**
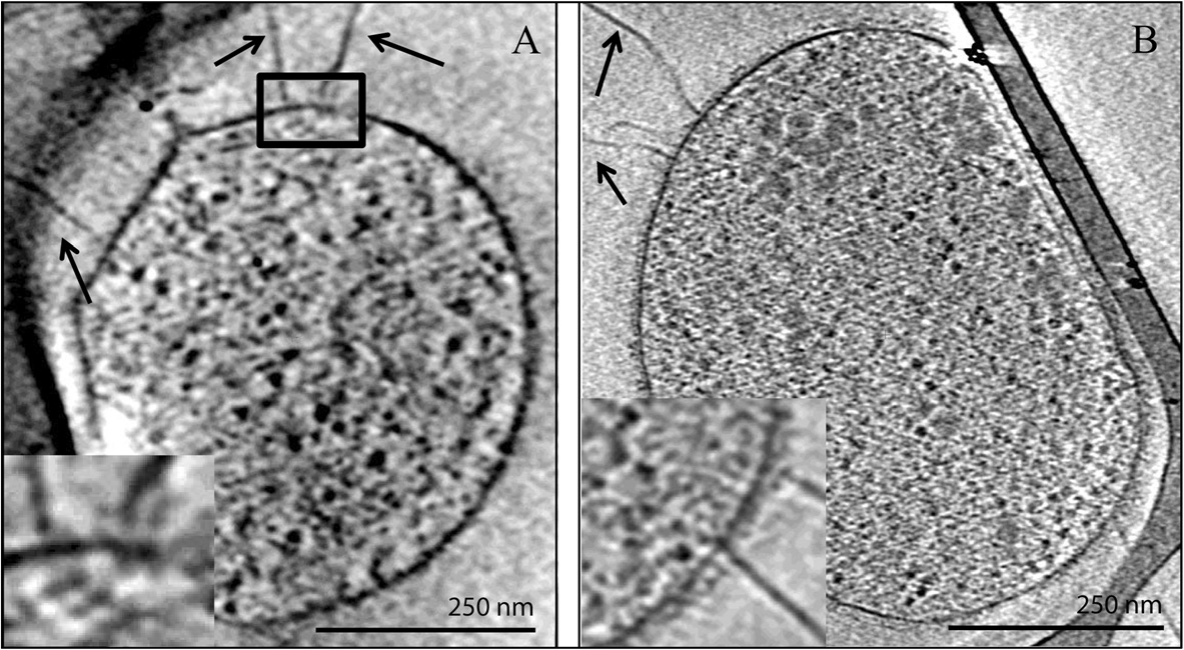
**Cryo-electron microscopy of AMD plasma cells.** Panel **A** and panel **B** show evidence of flagella on two different cells collected from the Richmond Mine AMD. Arrows point to flagella. The box surrounds a potential motor protein complex.

No flagellar genes are found in the other AMD plasma genomes, suggesting differences in motility. We used cryo-EM to confirm the existence of flagella on cells inferred to be archaea based on the presence of a single cell membrane (Figure 4). We found flagella-like structures with diameters of about 10–14 nm, similar in width to the flagella of *T. volcanium*[105]. The structures are also thicker than the pili observed in similar AMD plasmas or in bacteria [106]. A high-electron density area can be seen inside the cytoplasm immediately adjacent to the flagella that may be part of the associated protein motor complex.

**Figure 5:**
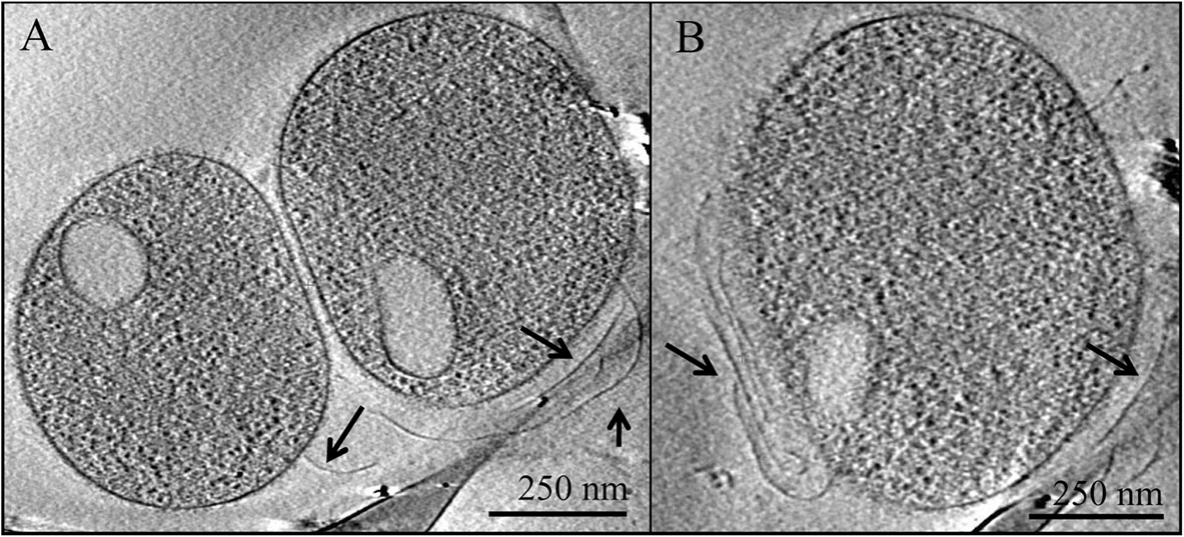
**Cryo-electron microscopy of AMD plasma cells with putative pili.** Panel **A** and panel **B** show evidence of pili on two different cells collected from the Richmond Mine AMD. Arrows point to pili. Vesicle-like structures are delineated by a single membrane layer around an ovoid shape in each cell’s cytoplasm.

In addition to flagellar assembly genes, a number of the AMD plasma genomes contain genes for Type II secretion or Type IV pili that are used in twitching motility or possibly conjugation or attachment to the biofilm or other surfaces. All of the genomes except for Fer1 and Fer2 contain some of these genes, and in Eplasma, Gplasma, and Iplasma they are in a cluster with conserved gene order among the AMD plasmas (Additional file 23). Cryo-EM confirms the existence of pili, and shows attachment of the pili from the original cell to other cells (Figure 5, Additional file 24).

### Vesicle-like cavities

Cryo-EM imaging demonstrates that a number of the AMD plasma cells harbor low electron-density inclusions within what appears to be a lipid membrane (Figure 5). These are similar in appearance to the gas vesicles that some extreme halophiles use for buoyancy [107], although those vesicles are enclosed in a proteinaceous membrane. We did not find genomic evidence of gas vesicle formation in the AMD plasmas by performing BLASTP searches of their genomes against the gas vesicle protein (gvp) genes of *Haloarchaea*[108]. Novel vesicle formation genes are expected and we speculate that these are liquid vesicles because their apparent lipid membrane would be gas-permeable.

## Conclusions

The metagenomic and phylogenetic analyses presented here reveal evolutionary, metabolic and cell structural differences among uncultivated archaea that occur in AMD biofilm communities. We recognize Iplasma as a representative of a phylogenetically distinct class and provide both ribosomal RNA gene-based and genomic evidence supporting this conclusion. We present evidence for two new genera of the *Thermoplasmatales* order (one comprising E- and Gplasma and another including A-, B-, C-, and Dplasma). Based on genome content, it appears that all of the AMD plasmas have the capacity to grow both aerobically and anaerobically. However, their differing genetic potentials for biosynthesis of cofactors and amino acid precursors may allow the coexisting AMD plasmas to take advantage of microniches that occur in structurally differentiated biofilms [87]. Similarly, differences in motility may allow some AMD plasmas to colonize new sites or move along physicochemical gradients. We report new types of blue-copper proteins that future work may show are involved in iron oxidation and may further differentiate the AMD plasmas. Comparative genomic analyses also provide new information about organisms in the *Thermoplasmatales* clade, indicating the importance of methylotrophy, carbon monoxide oxidation, and other heterotrophic metabolisms to the AMD plasmas and demonstrating the existence of S-layer proteins outside of the *Picrophilus* genus.

## Methods

### DNA sequencing and assembly

The new genomes presented here are composite assemblies of DNA extracted from a number of biofilm samples from the Richmond Mine, Iron Mountain, CA. Sample collection, DNA extraction, sequencing, genome assembly, and automated annotation were described previously [16,55,109,110], though current assemblies of Aplasma and Gplasma have been updated with recently acquired Illumina sequencing. All of the genomes were automatically assembled using velvet [111] and then manually curated, using the Consed software [112] to correct misassemblies and join contigs across gaps. Assembly data were published in Yelton, *et al*., 2011 [16].

### Gene annotation

In addition to the automated annotation pipeline for the genomes described [16], we used a synteny-based method to improve the annotations of poorly annotated genes. This method was described previously [16], and provides either specific or general functional annotations based on gene context in distantly related genomes.

We manually curated all annotations that are specifically cited in this paper in the following manner. Genes were aligned against the Interpro and nr databases with a BLASTP algorithm. Genes were then annotated if they had a TIGR or Pfam domain hit that predicted a specific function with an e-value of at least 1 × 10^-10^ and coverage of more than 70% of the protein. Genes were given a “putative” annotation if they met the previous criteria except they had an e-value between 1 × 10^-4^ and 1 × 10^-10^ and matched 50-70% of the protein, or if their domain-based hits provided only general functional information. In these cases, additional evidence from hits from the nr database was used if possible to provide a specific functional annotation. Genes were given a “probable” annotation if they had annotated hits in the nr database with greater than 30% amino acid identity over 70% of the length of the gene. For incomplete metabolic and structural pathways, BLASTP searches were carried out against the entire Richmond Mine metagenomic database. Missing genes were searched for based on the amino acid sequence of their closest relative. In the case where significant hits were uncovered, maximum-likelihood amino acid trees were used to place these genes within the AMD plasma group of archaea and this placement was used to associate the genes with a specific AMD plasma genome or outside the group altogether.

### Phylogenetic analyses

Phylogenetic analyses of certain genes were used to help place them in evolutionary context (e.g. 16S rRNA, blue-copper proteins). In these cases, the genes were aligned using the MAFFT alignment tool and default parameters [113,114]. The alignment was then manually corrected if needed. For protein trees, the completed alignment was used to make a phylogenetic tree with the FastTree [115,116] maximum likelihood-based tree software. In the case of the 16S rRNA gene, the phylogenetic tree was made using RaxML for improved accuracy based on the taxonomy of isolate organisms [117]. Support values were calculated for each branch split via the Shimodaira-Hasegawa test provided by the –boot option set to 1000 bootstraps for FastTree trees and using the rapid bootstrap for the RaxML tree.

### Cryo-EM specimen preparation

For cryo-EM, aliquots of 5 μl were taken directly from the fresh biofilm samples and placed onto lacey carbon grids (Ted Pella 01881) that were pre-treated by glow-discharge. For cryo-ET, samples were deposited onto support grids pre-loaded with 10 nm colloidal gold particles. The Formvar support was not removed from the lacey carbon. The grids were manually blotted and plunged into liquid ethane by a compressed air piston, then stored in liquid nitrogen.

### Electron tomography imaging

Images were acquired on a JEOL–3100 electron microscope equipped with a FEG electron source operating at 300 kV, an Omega energy filter, a Gatan 795 2K×2K CCD camera, and cryo-transfer stage. The stage was cooled to 80 K with liquid nitrogen. For more information on imaging and analysis see Additional file 25.

### Availability of supporting data

The data sets supporting the results of this article are available in the NCBI repository.

Aplasma: This Whole Genome Shotgun project has been deposited at DDBJ/EMBL/GenBank under the accession ACXK00000000. The version described in this paper is version ACXK02000000. Eplasma: This Whole Genome Shotgun project has been deposited at DDBJ/EMBL/GenBank under the accession ACXL00000000. The version described in this paper is version ACXL02000000. Gplasma: This Whole Genome Shotgun project has been deposited at DDBJ/EMBL/GenBank under the accession ATDV00000000. The version described in this paper is version ATDV01000000. FER1: This isolate genome has been deposited at DDBJ/EMBL/GenBank under the accession AMD_IFERC00001. FER2: This Whole Genome Shotgun project has been deposited at DDBJ/EMBL/GenBank under the accession ATDU00000000. The version described in this paper is version ATDU01000000. Iplasma: This Whole Genome Shotgun project has been deposited at DDBJ/EMBL/GenBank under the accession ACXM00000000. The version described in this paper is version ACXM02000000. Additional data sets supporting the results of this article are included within the article and its additional files.

## Competing interests

The authors declare that they have no competing interests.

## Authors’ contributions

Genome assemblies were carried out by AY, VD, and JB BT called and annotated genes in these assemblies. AY manually curated these gene annotations and applied the synteny-based annotation method to provide more specific annotations. LC performed the cryo-EM. NJ provided insight into metabolic comparisons. AY did the comparisons between the genomes. CC did the protein structural alignments. The manuscript was written by AY and JB. All authors read and approved the manuscript.

## Supplementary Material

Additional file 1 Percent nucleotide identity of 16S rRNA genes in the AMD plasmas relative to one another. Click here for file

Additional file 2 **16S rRNA nucleotide identity for AMD Thermoplasmatales organisms and close relatives.** Note that all of the organisms in the first column except for *Aciduliprofundum boonei* are classified as *Thermoplasmatales*. Click here for file

Additional file 3 Ribosomal protein S15 tree of the AMD plasma archaea and their close relatives. Click here for file

Additional file 4 Average amino acid identity of shared orthologs between the AMD plasma genomes. Click here for file

Additional file 5 Percentage of shared orthologs between the AMD plasma genomes. Click here for file

Additional file 6 **Gene order conservation between the AMD plasma genomes.** Synt/Orth indicates the number of syntenous orthologs divided by the total number of orthologs. Click here for file

Additional file 7 **Average length of syntenous blocks of genes between the AMD plasma genomes.** Synt Block indicates the average number of genes of syntenous blocks of genes in each pairwise comparison. Click here for file

Additional file 8 **Estimate of genome completeness based on orthologous marker gene homologs.** Note that genome estimates of 100% are not exact. These genomes still contain gaps between contigs. Click here for file

Additional file 9 **Metabolic and structural features of the AMD plasma organisms.** The surface layer proteins are pink. Pili are blue. Flagella are brown. The electron transport chain is yellow. The metal resistance proteins are blue. The archaeal type ATP synthase is yellow. Sulfocyanin is yellow and rusticyanin is blue. Click here for file

Additional file 10 **Cluster of unique genes in Gplasma.** PUF indicates a protein of unknown function. Bold font indicates gene numbers for proteins detected in proteomic data. Click here for file

Additional file 11 Cryo-EM movie of AMD plasma cell with S-layer proteins. Click here for file

Additional file 12 **Genes of metabolic and structural importance in the AMD plasma genomes.** * indicates a putative annotation. ** indicates a probable annotation. *** indicates a possible annotation. Gray indicates additional evidence of function via synteny analysis. Bold font indicates gene numbers for proteins detected in proteomic data. “split” indicates a split gene. “fusion” indicates a fused gene. Click here for file

Additional file 13 **Structural alignment of blue copper proteins.** β-Strands (cupredoxin fold) predicted by YASPIN [118] are highlighted (cyan for β-strand 1, yellow and light green for β-strand 2, pink for β-strand 3, dark blue for β-strand 4, dark green for β-strand 5, purple for β-strand 6 and red for β-strand 7). Amicyanin from *Paracoccus denitrificans* [GenBank: CAA39199] and Plastocyanin from *Synechococcus elongatus* GenBank: ABB57 [118] serve as references. Red circles indicate copper-binding ligands. Residues highlighted by light grey correspond to additional β-strands and those in bold orange correspond to α-helices. Sulfocyanin-specific motifs are boxed in red. Black arrows indicate copper-binding ligands. Additional loops are indicated at the bottom of the alignment by a light orange line. Click here for file

Additional file 14 Blue-copper protein motifs found in AMD plasma genes. Click here for file

Additional file 15 **AMD plasma blue-copper protein tree.** bcp indicates a blue-copper protein of unknown function. Click here for file

Additional file 16 AMD plasma CODH gene tree. Click here for file

Additional file 17 **Active site alignment of aerobic CODH catalytic subunit genes.** The red box indicates the active site residues. *H. pseudoflava* is *Hydrogenophaga pseudoflava*, *O. carboxidovorans* is *Oligotropha carboxidovorans*, *M. loti* is *Mesorhizobium loti*, *B. japonicum* is *Bradyrhizobium japonicum*, and *B. fungorum* is *Burkholderia fungorum*. Click here for file

Additional file 18 **Ni-CODH catalytic subunit alignment.** Genes in this alignment are the Ni-CODH catalytic subunits from *R. rubrum* (CooS, PDB:1JQK), *M. thermoacetica* (AcsA, PDB:1MJG) and Fer2 (fer2_31_0047). fer2_31_0047’s secondary structure was predicted by YASPIN [118]. β-strands are shown in green and α-helices are highlighted in cyan. Residues belonging to the D-cluster are boxed in yellow (Cys41 and Cys49). Ligands of the B-cluster are boxed in black (Cys50, Cys53, Cys58 and Cys72). Catalytic residues binding the Ni-Fe-S cluster from C-cluster are boxed in purple (His265, Cys300, Cys338, Cys451, Cys481, and Cys531) and catalyze the oxidation of carbon. His95 and Lys568 (boxed in dark red) are non-coordinating residues conserved in Ni-CODHs and have been suggested to be involved in facilitating the reaction [119]. Residue numbering is from the *R. rubrum* Ni-CODH. Click here for file

Additional file 19 **Cytochrome c oxidase subunit II alignment.** * indicates the copper-binding motif found in other cytochrome c oxidase proteins. *S. acidocaldarius* is *Sulfolobus acidocaldarius*, *A. pernix* is *Aeropyrum pernix*, *P. oguniense* is *Pyrobaculum oguniense*, *T. thermophilus* is *Thermus thermophilus*, *P. denitrificans* is *Paracoccus denitrificans*. Click here for file

Additional file 20 Amino acid identity of AMD plasma cytochrome c oxidase subunit II genes with closely related genes. Click here for file

Additional file 21 **AMD plasma gene homologs to genes overexpressed or overtranscribed under anaerobic conditions in *T. volcanium *and *T. acidophilum ***[75]**,**[76]**.** Bold font indicates gene numbers for proteins detected in proteomic data. Click here for file

Additional file 22 **AMD plasma putative hydrogenase 4 gene tree.** Accession numbers are to the left of the species names. Click here for file

Additional file 23 **Pili genes in the AMD plasmas.** * indicates a putative annotation. ** indicates a probable annotation. *** indicates a possible annotation. Gray indicates additional evidence of function via synteny analysis. “split” indicates a split gene. Bold font indicates gene numbers for proteins detected in proteomic data. Click here for file

Additional file 24 Cryo-EM movie of AMD plasma cells with flagella, pili, and viruses. Click here for file

Additional file 25 Additional information on cryo-EM imaging. Click here for file
