# Supplementary figures and images for: Comparative genomics in acid mine drainage biofilm communities reveals metabolic and structural differentiation of co-occurring archaea
Source: BMC Genomics. 2013 Jul 17;14:485. doi: 10.1186/1471-2164-14-485 (PMC3750248; doi:10.1186/1471-2164-14-485)

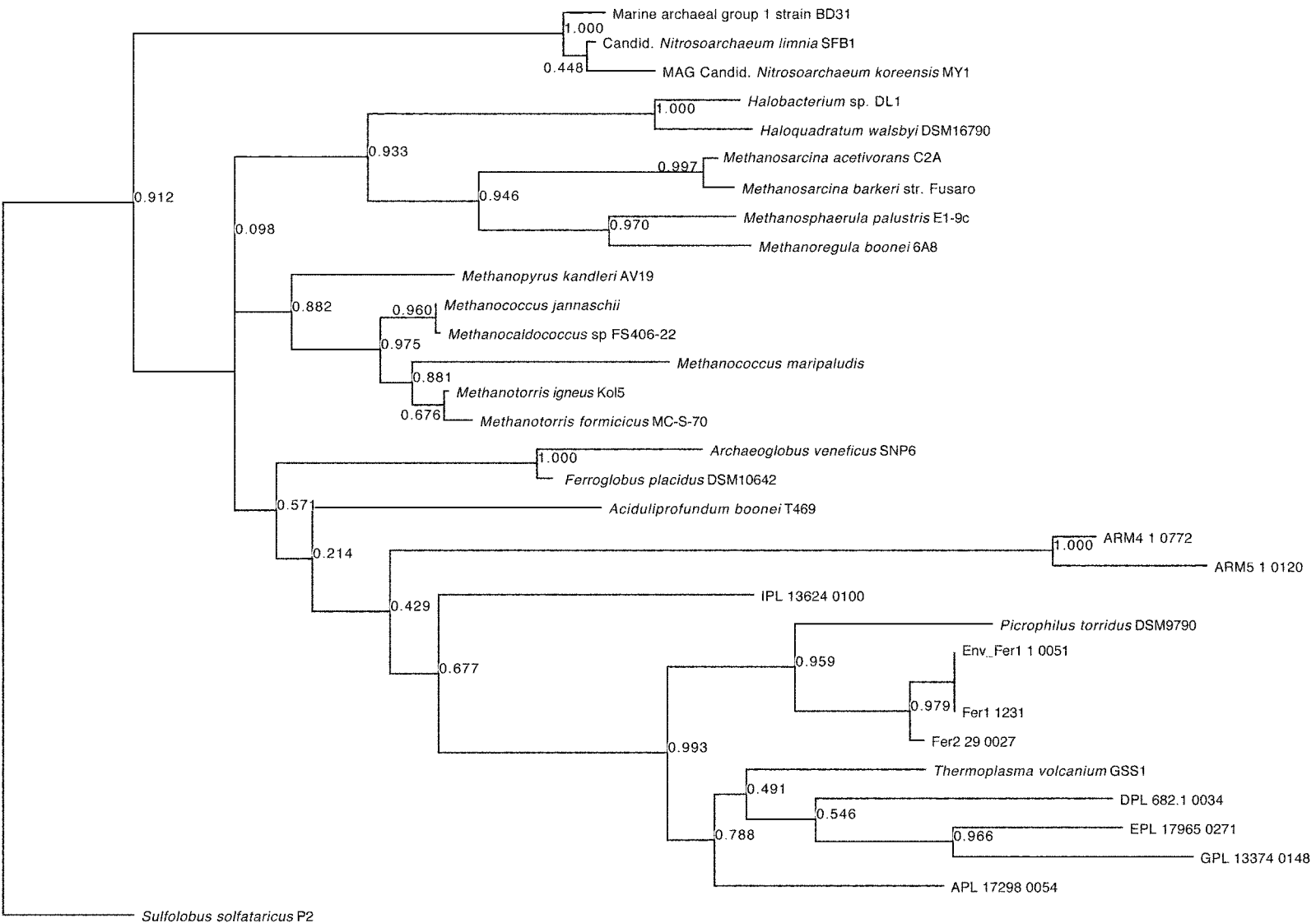

0.10

Supplement: Additional file 3 — Ribosomal protein S15 tree of the AMD plasma archaea and their close relatives. [file 1471-2164-14-485-S3.pdf]

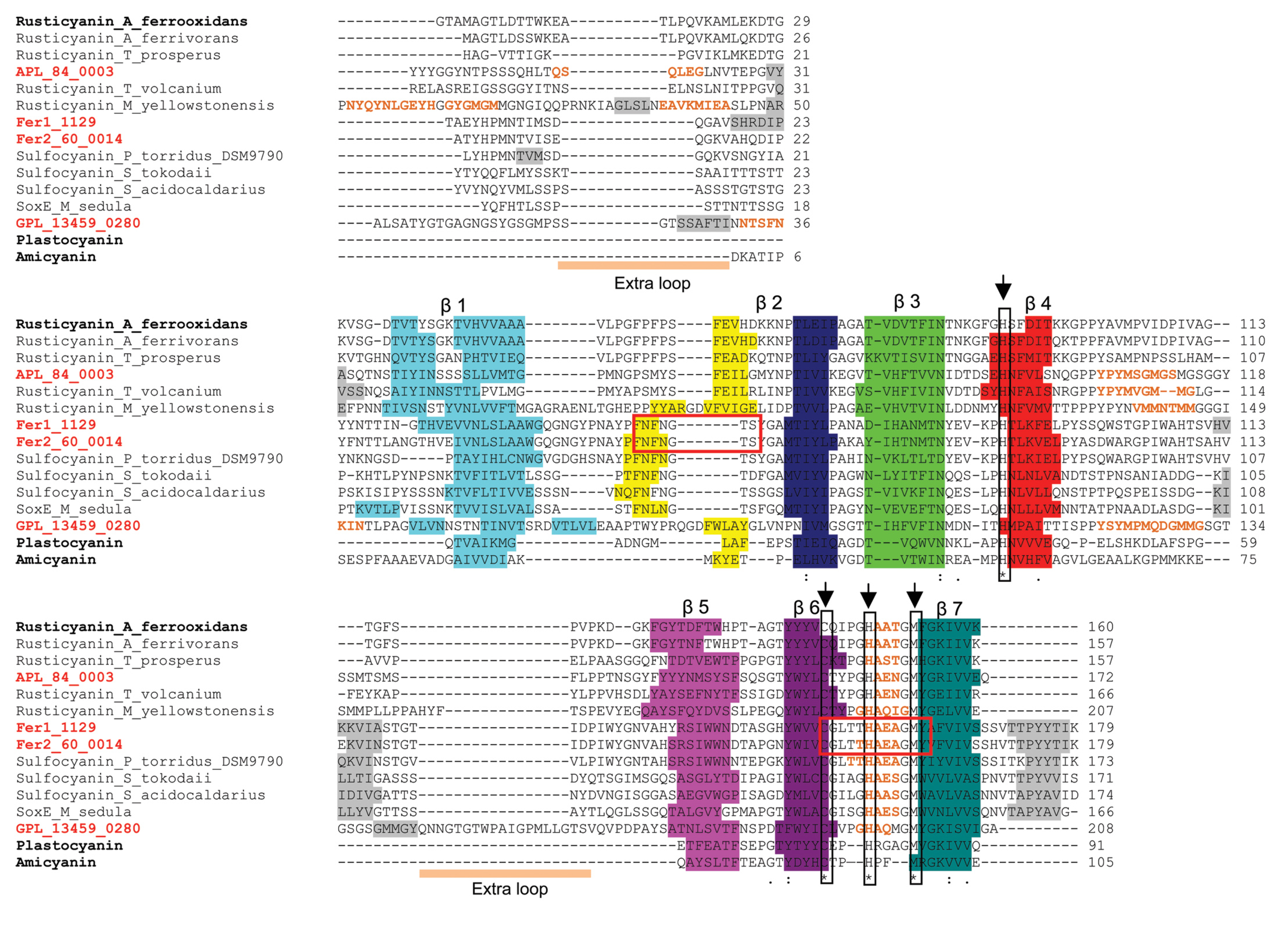

Supplement: Additional file 13 — Structural alignment of blue copper proteins. β-Strands (cupredoxin fold) predicted by YASPIN [118] are highlighted (cyan for β-strand 1, yellow and light green for β-strand 2, pink for β-strand 3, dark blue for β-strand 4, dark green for β-strand 5, purple for β-strand 6 and red for β-strand 7). Amicyanin from Paracoccus denitrificans [GenBank: CAA39199] and Plastocyanin from Synechococcus elongatus GenBank: ABB57 [118] serve as references. Red circles indicate copper-binding ligands. Residues highlighted by light grey correspond to additional β-strands and those in bold orange correspond to α-helices. Sulfocyanin-specific motifs are boxed in red. Black arrows indicate copper-binding ligands. Additional loops are indicated at the bottom of the alignment by a light orange line. [file 1471-2164-14-485-S13.jpeg]

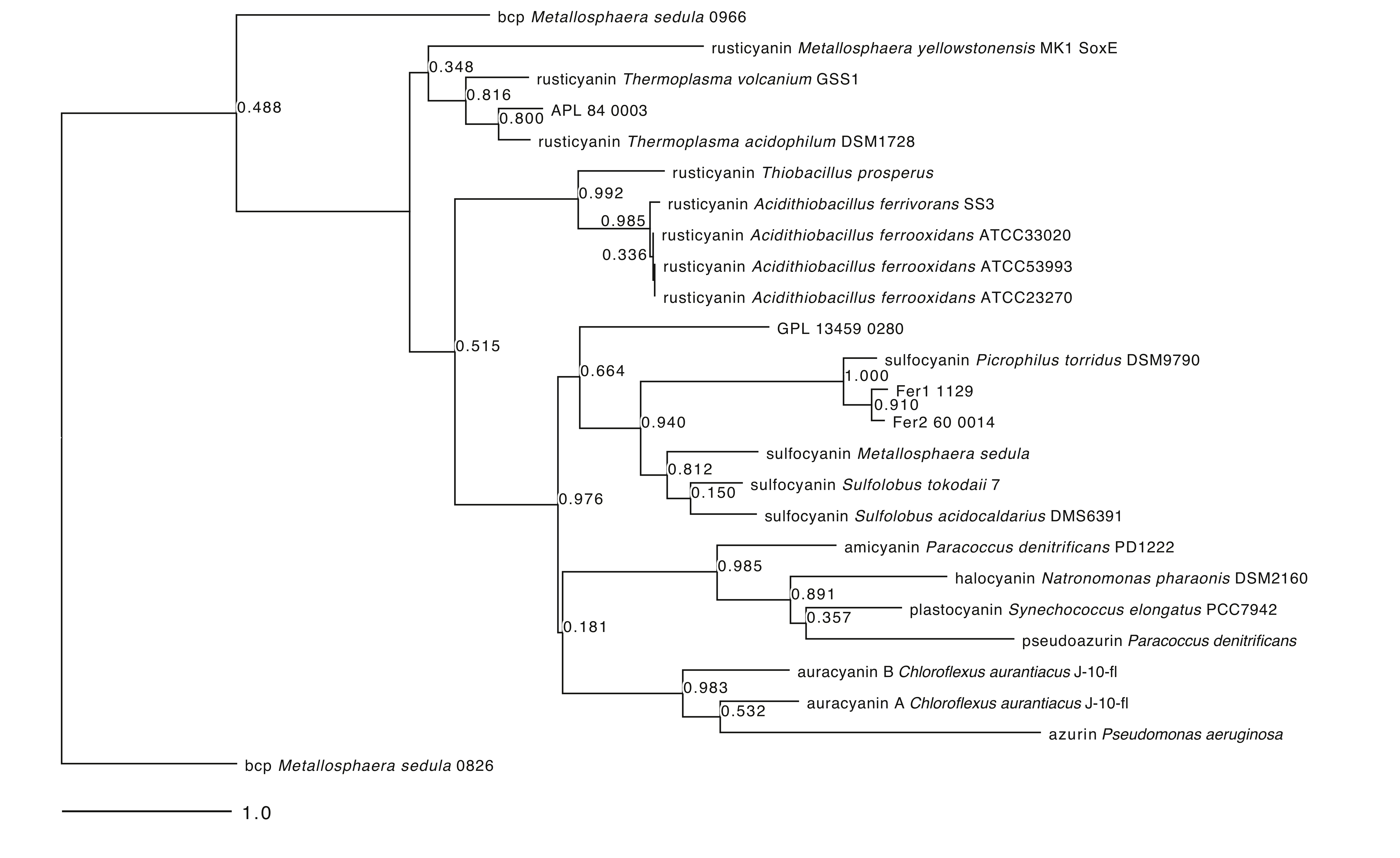

Supplement: Additional file 15 — AMD plasma blue-copper protein tree. bcp indicates a blue-copper protein of unknown function. [file 1471-2164-14-485-S15.jpeg]

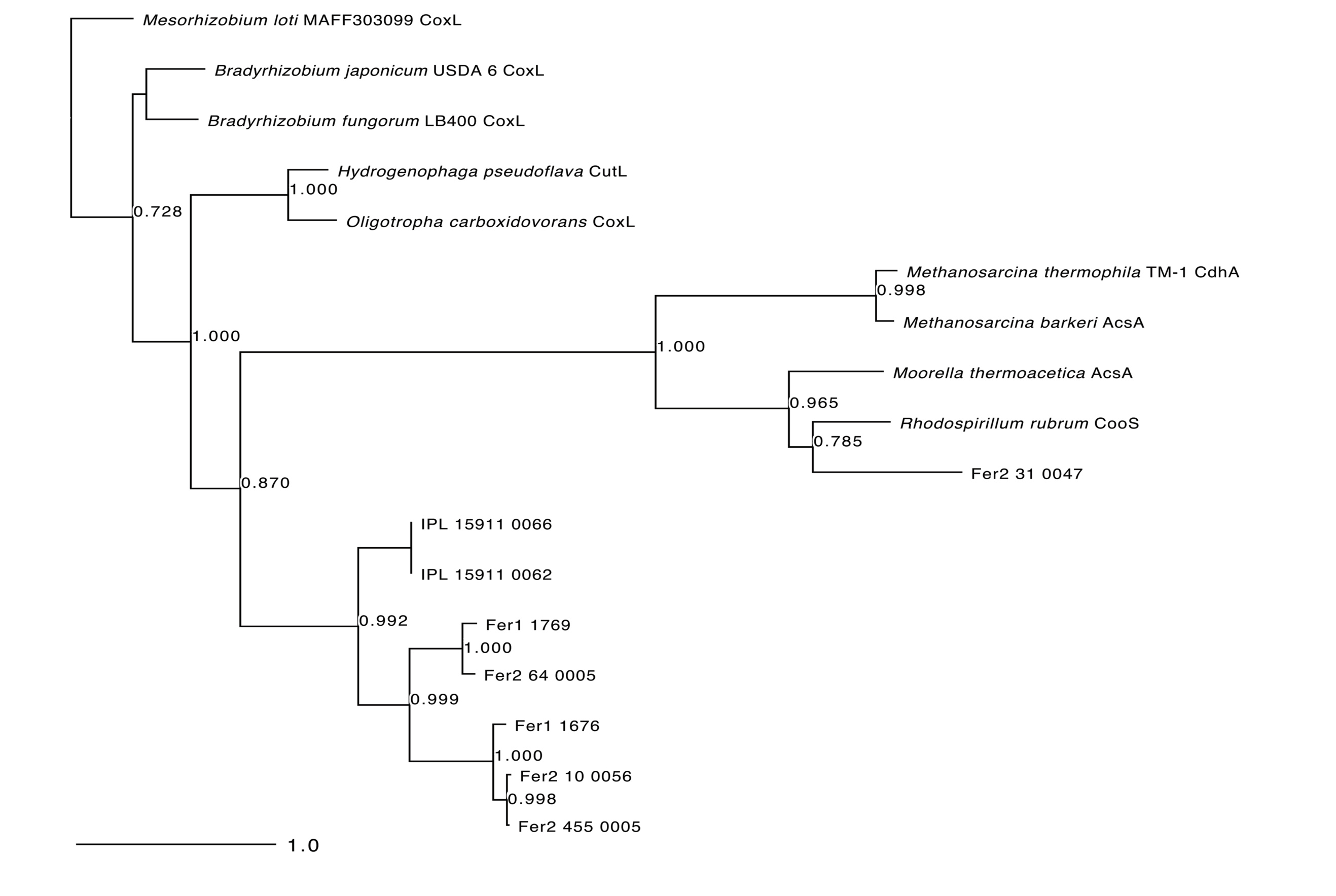

Supplement: Additional file 16 — AMD plasma CODH gene tree. [file 1471-2164-14-485-S16.jpeg]

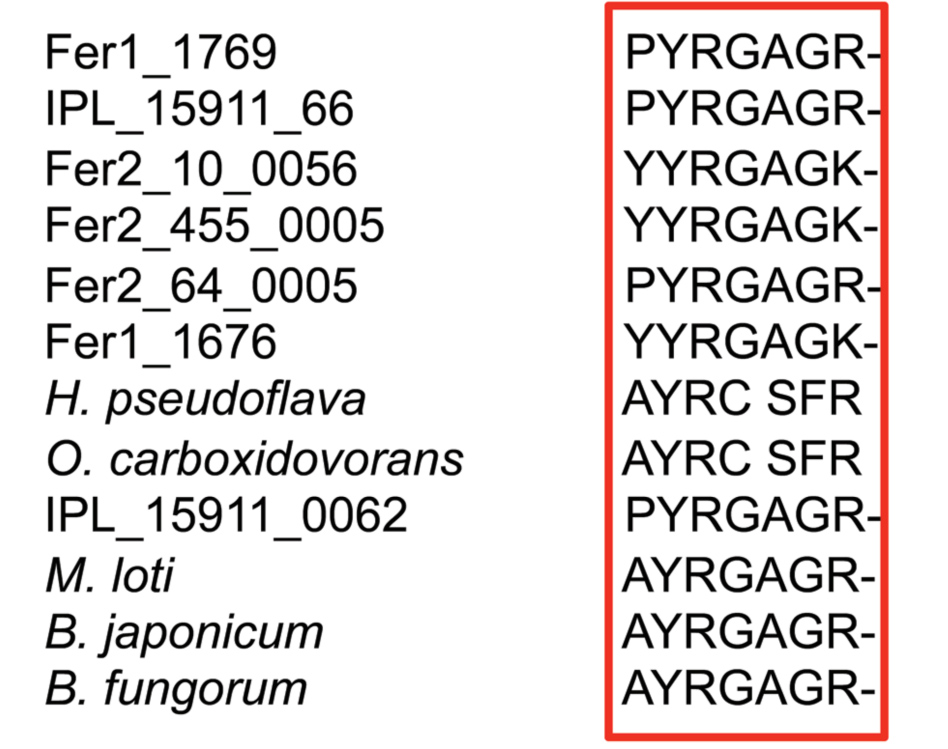

Supplement: Additional file 17 — Active site alignment of aerobic CODH catalytic subunit genes. The red box indicates the active site residues. H. pseudoflava is Hydrogenophaga pseudoflava, O. carboxidovorans is Oligotropha carboxidovorans, M. loti is Mesorhizobium loti, B. japonicum is Bradyrhizobium japonicum, and B. fungorum is Burkholderia fungorum. [file 1471-2164-14-485-S17.jpeg]

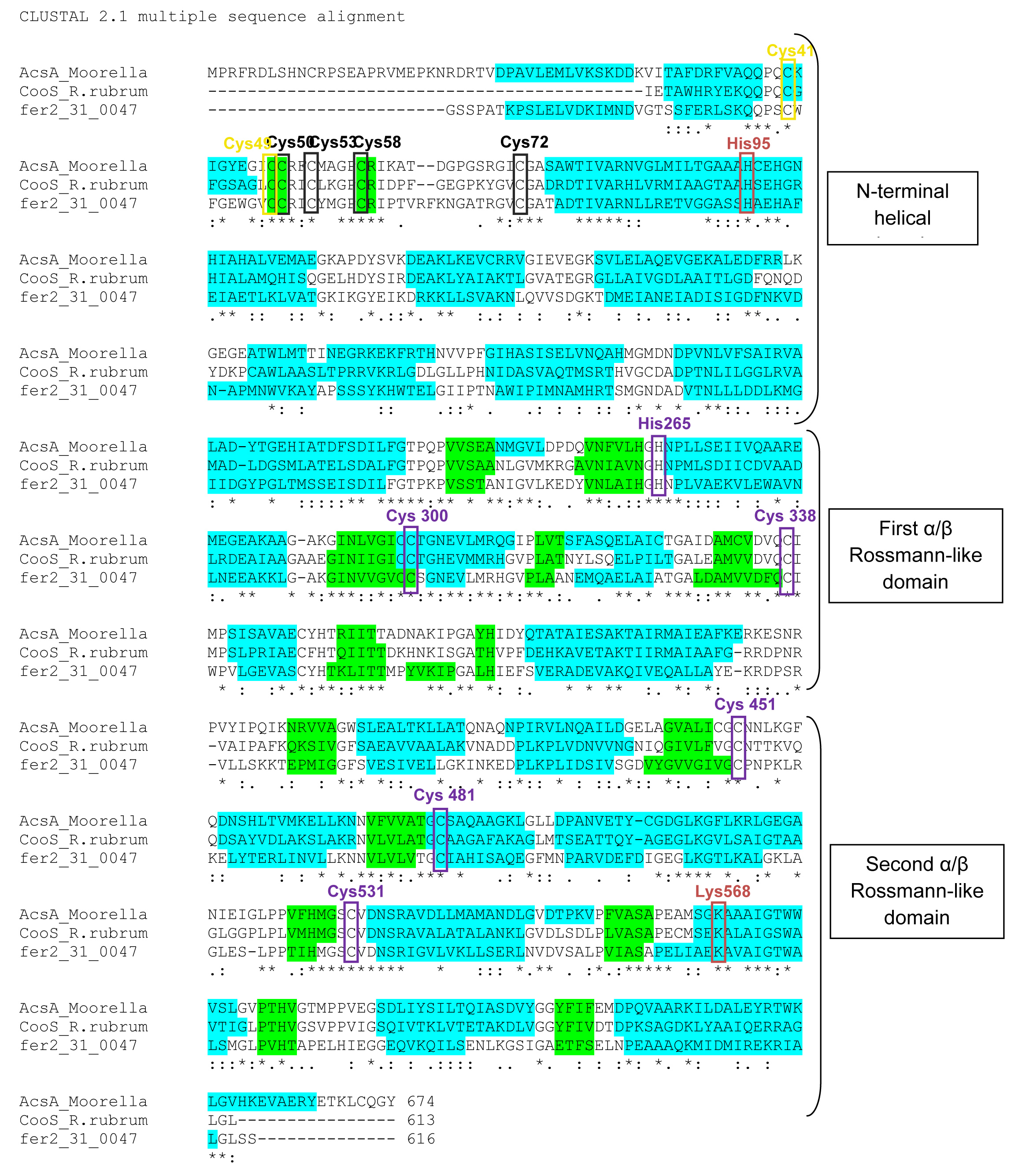

Supplement: Additional file 18 — Ni-CODH catalytic subunit alignment. Genes in this alignment are the Ni-CODH catalytic subunits from R. rubrum (CooS, PDB:1JQK), M. thermoacetica (AcsA, PDB:1MJG) and Fer2 (fer2_31_0047). fer2_31_0047’s secondary structure was predicted by YASPIN [118]. β-strands are shown in green and α-helices are highlighted in cyan. Residues belonging to the D-cluster are boxed in yellow (Cys41 and Cys49). Ligands of the B-cluster are boxed in black (Cys50, Cys53, Cys58 and Cys72). Catalytic residues binding the Ni-Fe-S cluster from C-cluster are boxed in purple (His265, Cys300, Cys338, Cys451, Cys481, and Cys531) and catalyze the oxidation of carbon. His95 and Lys568 (boxed in dark red) are non-coordinating residues conserved in Ni-CODHs and have been suggested to be involved in facilitating the reaction [119]. Residue numbering is from the R. rubrum Ni-CODH. [file 1471-2164-14-485-S18.jpeg]

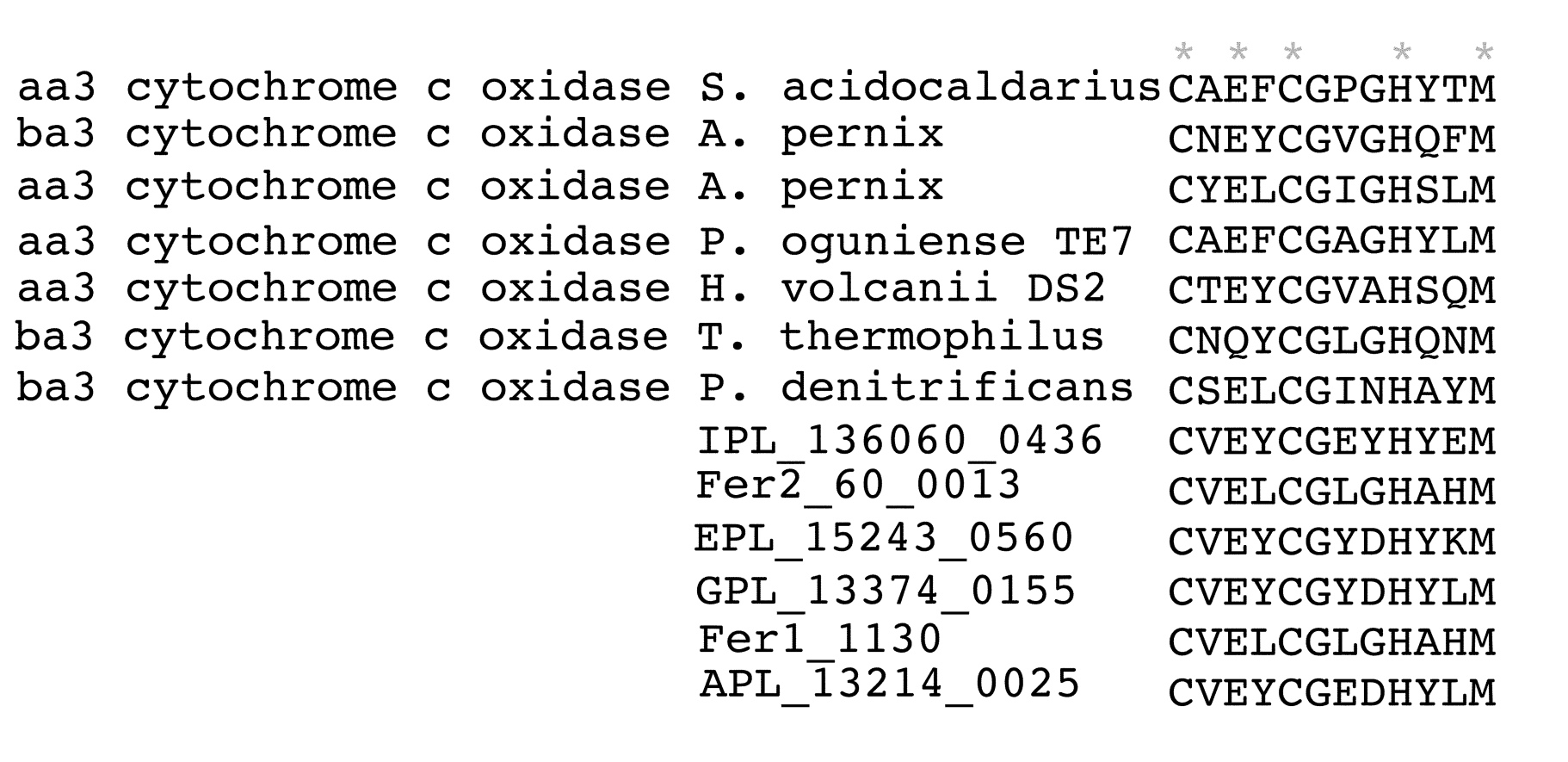

Supplement: Additional file 19 — Cytochrome c oxidase subunit II alignment. * indicates the copper-binding motif found in other cytochrome c oxidase proteins. S. acidocaldarius is Sulfolobus acidocaldarius, A. pernix is Aeropyrum pernix, P. oguniense is Pyrobaculum oguniense, T. thermophilus is Thermus thermophilus, P. denitrificans is Paracoccus denitrificans. [file 1471-2164-14-485-S19.jpeg]

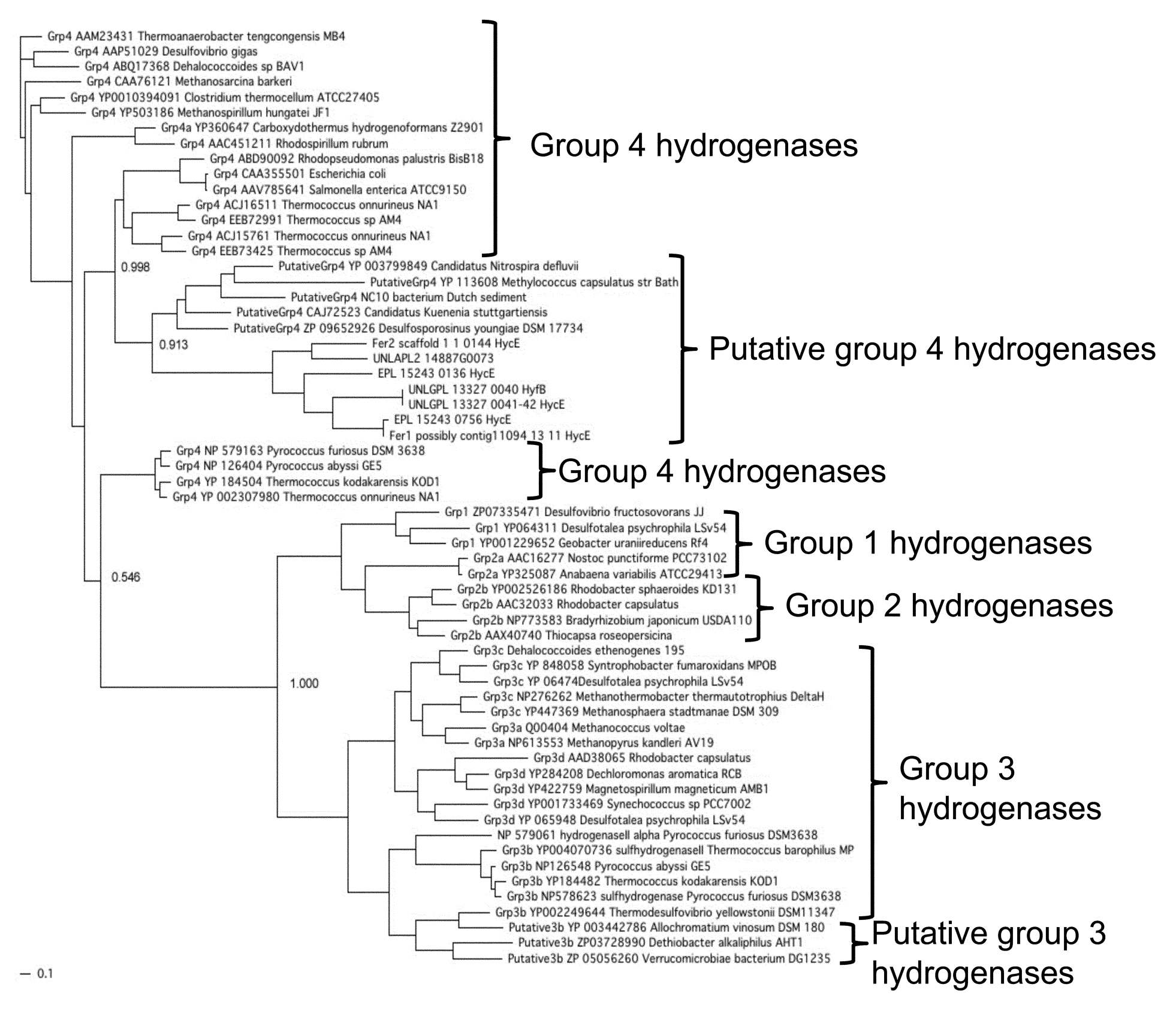

Supplement: Additional file 22 — AMD plasma putative hydrogenase 4 gene tree. Accession numbers are to the left of the species names. [file 1471-2164-14-485-S22.jpeg]
