# Supplementary material for: Comparative genomics in acid mine drainage biofilm communities reveals metabolic and structural differentiation of co-occurring archaea
Source: BMC Genomics. 2013 Jul 17;14:485. doi: 10.1186/1471-2164-14-485 (PMC3750248; doi:10.1186/1471-2164-14-485)
Supplement: Additional file 25 — Additional information on cryo-EM imaging. [file 1471-2164-14-485-S25.doc]

**Additional information on cryo-EM imaging.**

The data presented here is part of a large dataset comprising over 800 images or 2D projections and 69 tilt series recorded using magnifications of 36K, 30Kx, and 25Kx at the CCD giving a pixel size of 0.83 nm, 1.0 nm or 1.2 nm, respectively. Underfocus values ranged from 8 µm ± 0.5 um to 14 µm ± 0.5 µm, and energy filter widths were approximately 22 eV ± 2 eV.

Tomographic tilt series were acquired under low-dose conditions, typically over an angular range of +65 deg to -65 deg, ± 5 deg with increments of 1° or 2°. 70 to 124 images were recorded for each series. Tilt series were acquired semi-automatically with the program SerialEM adapted to JEOL microscopes. For all datasets the maximum dose used per complete tilt series was approximately 150 e-/Å2, with a typical value of 100 e-/Å2.

The software used for image analysis are as follows: Imod for tomographic reconstructions , ImageJ (NIH, http://rsb.info.nih.gov/ij/) for analysis of the two-dimensional image projections, VisIt (<http://www.llnl.gov/visit>) for volume rendering and image analysis of tomographic reconstructions, and the ffmpeg package ([www.ffmpeg.org](http://www.ffmpeg.org/)) to make all movies.
